# The jumping to conclusions reasoning bias as a cognitive factor contributing to psychosis progression and persistence: findings from NEMESIS-2

**DOI:** 10.1017/S0033291720000446

**Published:** 2021-07

**Authors:** Christian Rauschenberg, Ulrich Reininghaus, Margreet ten Have, Ron de Graaf, Saskia van Dorsselaer, Claudia J. P. Simons, Nicole Gunther, Cécile Henquet, Lotta-Katrin Pries, Sinan Guloksuz, Maarten Bak, Jim van Os

**Affiliations:** 1 Department of Psychiatry and Neuropsychology, School for Mental Health and Neuroscience, Maastricht University, Maastricht, The Netherlands; 2 Department of Public Mental Health, Central Institute of Mental Health, Medical Faculty Mannheim, University of Heidelberg, Mannheim, Germany; 3 Health Service and Population Research Department, Centre for Epidemiology and Public Health, Institute of Psychiatry, Psychology & Neuroscience, King's College London, London, UK; 4 Department of Epidemiology, Netherlands Institute of Mental Health and Addiction, Utrecht, The Netherlands; 5 GGzE, Institute for Mental Health Care Eindhoven and De Kempen, Eindhoven, The Netherlands; 6 School of Psychology, Open University, Heerlen, The Netherlands; 7 Department of Psychiatry, Yale University School of Medicine, New Haven, CT, USA; 8 Department of Psychiatry, Brain Centre Rudolf Magnus, University Medical Centre Utrecht, Utrecht, The Netherlands; 9 Department of Psychosis Studies, Institute of Psychiatry, Psychology & Neuroscience, King's College London, London, UK

**Keywords:** Cognitive models, jumping to conclusions, persistence, progression, psychosis, psychotic experiences, reasoning bias, transdiagnostic phenotype

## Abstract

**Background:**

Contemporary models of psychosis implicate the importance of affective dysregulation and cognitive factors (e.g. biases and schemas) in the development and maintenance of psychotic symptoms, but studies testing proposed mechanisms remain limited. This study, uniquely using a prospective design, investigated whether the jumping to conclusions (JTC) reasoning bias contributes to psychosis progression and persistence.

**Methods:**

Data were derived from the second Netherlands Mental Health Survey and Incidence Study (NEMESIS-2). The Composite International Diagnostic Interview and an add-on instrument were used to assess affective dysregulation (i.e. depression, anxiety and mania) and psychotic experiences (PEs), respectively. The beads task was used to assess JTC bias. Time series analyses were conducted using data from T1 and T2 (*N* = 8666), excluding individuals who reported high psychosis levels at T0.

**Results:**

Although the prospective design resulted in low statistical power, the findings suggest that, compared to those without symptoms, individuals with lifetime affective dysregulation were more likely to progress from low/moderate psychosis levels (state of ‘aberrant salience’, one or two PEs) at T1 to high psychosis levels (‘frank psychosis’, three or more PEs or psychosis-related help-seeking behaviour) at T2 if the JTC bias was present [adj. relative risk ratio (RRR): 3.8, 95% confidence interval (CI) 0.8–18.6, *p* = 0.101]. Similarly, the JTC bias contributed to the persistence of high psychosis levels (adj. RRR: 12.7, 95% CI 0.7–239.6, *p* = 0.091).

**Conclusions:**

We found some evidence that the JTC bias may contribute to psychosis progression and persistence in individuals with affective dysregulation. However, well-powered prospective studies are needed to replicate these findings.

## Introduction

Psychosis spectrum disorders have a complex aetiology and multifaceted phenomenology. Psychotic experiences (PEs), the attenuated subclinical expression of positive psychotic symptoms, are common, with an estimated lifetime prevalence ranging from 5% to 8% (van Os & Reininghaus, 2016). PEs are often preceded by, or co-occur with, affective dysregulation (e.g. depression and anxiety) (Wigman et al., 2012), which is in accordance with clinical observations of frequent comorbidity of affective disorders with psychotic disorders as well as substantially overlapping genetic liability (Brainstorm Consortium et al., 2018). These findings have been taken to suggest a transdiagnostic and extended psychosis phenotype with temporal and phenomenological continuity across the developmental stages of psychotic and affective disorders (van Os & Reininghaus, 2016). Importantly, however, PEs are often transitory and neither inherently distressing nor inevitably impairing (Loberg, Gjestad, Posserud, Kompus, & Lundervold, 2019; Wusten et al., 2018), but persist in around 20% (Linscott & van Os, 2013). Of these, approximately 7% develop a psychotic disorder (Linscott & van Os, 2013). Consequently, there has been an increasing interest in identifying clinically informative predictors for illness onset (Fusar-Poli et al., 2019; Hartmann, Nelson, Ratheesh, Treen, & McGorry, 2019; Nelson, McGorry, Wichers, Wigman, & Hartmann, 2017).

Contemporary models of psychosis implicate the importance of cognitive factors (e.g. schemas and biases) in illness trajectories by contributing to transforming experiences of aberrant salience into frank psychosis as well as symptom persistence (Freeman, 2016; Howes & Murray, 2014). Specifically, individuals' appraisal of, and response to (Garety & Freeman, 1999; Peters et al., 2017; Ward, Garety, Jackson, & Peters, 2019), these excessively vivid and intense experiences, which may be mediated by dysregulated dopaminergic signalling (Howes & Murray, 2014; Howes, McCutcheon, Owen, & Murray, 2017), are thought to be integral for the development of psychosis-related distress and impairment (Freeman, 2016). The interpretation of experiences as threatening and of external causation (Freeman, 2016; Garety, Bebbington, Fowler, Freeman, & Kuipers, 2007; Garety, Kuipers, Fowler, Freeman, & Bebbington, 2001; Howes & Murray, 2014), combined with altered behaviour and cognitive responses, has been shown to be associated with psychosis spectrum disorders (Brown, Waite, & Freeman, 2019; Ward et al., 2019).

The jumping to conclusions (JTC) reasoning bias is among the most widely studied cognitive biases in psychosis and describes individuals' tendency to make hasty decisions based on insufficient information (Dudley, Taylor, Wickham, & Hutton, 2016). It has been consistently found to be associated with subclinical and clinical psychosis (Dudley et al., 2016; Fine, Gardner, Craigie, & Gold, 2007; Ross, McKay, Coltheart, & Langdon, 2015; So, Siu, Wong, Chan, & Garety, 2016) as well as a transdiagnostic phenotype of co-occurring affective dysregulation and PEs (Reininghaus et al., 2019), but not with non-psychotic disorders (So et al., 2016). Thus, the JTC bias may be an important cognitive factor involved in the formation and maintenance of psychosis, especially delusional ideations (Brown et al., 2019; Freeman & Garety, 2014), with some recent meta-analytical evidence supporting this assumption (Dudley et al., 2016). Critically, most studies have investigated associations of JTC bias with psychosis using cross-sectional designs (So et al., 2016) and evidence on proposed specificity of JTC bias with psychosis in individuals with a transdiagnostic phenotype of mental health problems remains very limited (Reininghaus et al., 2019).

Longitudinal studies investigating the role of JTC bias in psychosis are rare. These studies suggest that JTC bias may be (i) predictive for less improvement of psychotic symptoms over time (Rodriguez et al., 2018), (ii) stable despite symptom remission (Peters & Garety, 2006; So et al., 2012), (iii) associated with poorer outcomes in people with psychosis (Dudley et al., 2013) as well as antipsychotic treatment response (Menon, Mizrahi, & Kapur, 2008), and (iv) associated with worse vocational functioning (Andreou et al., 2014). To our knowledge, no study has directly and prospectively tested the contribution and specificity of JTC bias to psychosis progression and persistence in individuals with co-occurring affective dysregulation, as proposed in recent integrative socio-developmental-cognitive models (Howes & Murray, 2014).

### Aims and hypotheses

For the current study, we aimed to extend previous research by investigating whether the JTC bias contributes to psychosis progression and persistence in the general population. Specifically, the following hypotheses were tested: first, compared to individuals with neither affective dysregulation nor PEs, the JTC bias is associated with an increased risk to progress from low/moderate psychosis levels (i.e. one or two PEs; hereafter ‘state of aberrant salience’) at T1 to high psychosis levels (i.e. three or more PEs or psychosis-related help-seeking behaviour; hereafter ‘frank psychosis’) at T2 in individuals with co-occurring lifetime affective dysregulation. Second, the JTC bias does not contribute to first-occurrence of frank psychosis at T2 when individuals report the sole presence of affective dysregulation at T1. Third, the JTC bias is associated with an increased risk to report persistence of frank psychosis between T1 and T2 in individuals with lifetime affective dysregulation.

## Materials and method

### Sample

Data were obtained from the second Netherlands Mental Health Survey and Incidence Study (NEMESIS-2), a nationally representative cohort study designed to investigate the incidence, prevalence, course and outcome of mental disorders. For current analyses, we used data of the first three waves (T0–T2), while, in the meantime, the fourth wave (T3) has been completed. A multistage, stratified random sampling of households was applied to ensure representativeness of the sample in terms of age, region and population density. First, a random sample of 184 of 443 municipalities was drawn, which were stratified by four regions (north, east, south and west) and five levels of population density. The four largest cities (i.e. Amsterdam, Rotterdam, The Hague and Utrecht) were also included. Thus, 24 strata were used to stratify the sample. The number of addresses selected per municipality was based on the distribution of the number of inhabitants aged 18–64 years. Second, a random sample of households was drawn from these addresses with the same probability to be selected. Finally, an individual aged 18–64 years was included based on the most recent birthday at first contact. The face-to-face interviews were performed at home by trained interviewers, who were not clinicians, using a laptop computer. The Composite International Diagnostic Interview (CIDI) version 3.0 (Kessler & Ustun, 2004) and additional questionnaires were used. The inclusion criterion was: aged 18–65 years. Exclusion criterion was insufficient command of the Dutch language. The first wave (T0) was conducted from November 2007 to July 2009 and enrolled 6646 participants (response rate: 65.1%). Although younger subjects were slightly underrepresented, this sample was representative for the Dutch general population (de Graaf, Ten Have, & van Dorsselaer, 2010). In the second wave (T1), carried out from November 2010 to June 2012, all respondents were approached, and, of these, 5303 individuals were interviewed again (response rate: 80.4% from T0 with those deceased excluded). From November 2013 to June 2015, the third wave (T2) was completed with 4618 persons who were interviewed a third time (response rate: 87.8% from T1 with those deceased excluded). Psychopathology reported at T0 (baseline) reflects lifetime prevalence, while symptoms reported at T1 and T2 reflect 3-year interval occurrence (i.e. between T0 and T1 as well as T1 and T2, respectively). Data from T1 and T2 were used for the current study. The Medical Ethics Review Committee for Institutions of Mental Health Care approved the study. More details on the study are provided elsewhere (de Graaf et al., 2010).

### Data collection

#### Socio-demographic characteristics and cognitive alterations

Data on age, sex and level of education were assessed in the additional questionnaire. The digit-span task, based on the Wechsler Adult Intelligence Scale (WAIS-III) (Wechsler, 1997), was used to assess working memory performance as a proxy for cognitive alterations.

#### JTC bias

The beads task, a probabilistic reasoning task, was completed at the third wave (T2) to assess the presence or the absence of JTC bias. The beads task is designed to measure individuals' reasoning style under ambiguous conditions (Phillips & Edwards, 1966). During the task, individuals were shown two jars with red and blue coloured beads in opposite ratios (i.e. 60 to 40 beads). The jars as well as all instructions were presented on a laptop screen. After a training session, participants were informed that the beads will be drawn consecutively from one jar and, once shown, returned to the same jar. After each draw, participants were asked whether they want to decide from which of the two jars the beads were drawn or if they prefer to see another bead. Although not communicated, participants were allowed to see up to 20 beads before they had to decide. The order of beads was fixed and the colour shown in the training session selected at random. The number of beads drawn at T2 was considered to represent individuals' trait reasoning style. Based on previous studies (Dudley et al., 2016), the presence of JTC bias was defined as making a decision based on two or fewer beads (So et al., 2016).

#### Affective dysregulation

Affective dysregulation was assessed at all three timepoints using the core items of CIDI version 3.0. The CIDI measure for core symptoms uses a true–false response format to screen for the prevalence of various mental disorders, including depressive episode, social phobia, generalised anxiety disorder and manic episode (e.g. feeling fearful, depressed and experiences of a panic attack). For current analyses, a variable was constructed that combines reported affective dysregulation (i.e. depression, anxiety and mania) from all assessment points (T0–T2). Thus, the variable affective dysregulation represents both lifetime prevalence (T0) as well as interval occurrence (T0–T1 and T1–T2) (binary variable). All items are presented in online Supplementary Table S1.

#### Psychotic experiences (PEs)

As earlier studies concluded that the CIDI is not a reliable and valid measures for psychotic disorders, a psychosis measure was constructed based on the psychosis section of the CIDI 1.1. The measure consisted of 20 items asking for PEs (i.e. 15 delusional and five hallucinatory experiences). At T0, the lifetime prevalence was assessed, while at T1 and T2, individuals were asked whether PEs have occurred between assessment points (i.e. interval occurrence). In case PEs were endorsed, participants were asked to state, on a 4-point Likert scale, the frequency, distress and the impact of PEs on their daily life, including whether they had sought help for these experiences. For current analyses, we used the number of PEs endorsed, irrespective of reported frequency, distress and impact. Sum scores were calculated by adding reported PEs. The used items are reported in online Supplementary Table S1. To determine psychosis-related help-seeking behaviour, the service assessments of the CIDI 3.0 were used.

#### Grouping absence, presence and co-occurrence of affective dysregulation and psychotic experiences

In line with previous work (Radhakrishnan et al., 2019; Reininghaus et al., 2019), individuals were grouped based on answers given on measures assessing depression, anxiety and mania (summarised as affective dysregulation) and PEs. Five groups were generated representing the absence, sole presence or co-occurrence of lifetime affective dysregulation and PEs: neither affective dysregulation nor PEs (group 1); the sole presence of affective dysregulation (group 2); the sole presence of PEs (group 3); affective dysregulation and aberrant salience (group 4; one or two PEs); affective dysregulation and frank psychosis (group 5; three or more PEs or psychosis-related help-seeking behaviour).

### Statistical analysis

All analyses were performed using STATA version 13.1 (StataCorp, 2013). First, individuals with frank psychosis and any affective dysregulation at T0 (lifetime prevalence) were excluded from analyses (*N* = 198), making sure incident states of frank psychosis at T1 and T2 were identifiable. Second, socio-demographic characteristics (i.e. age, sex and education level) and cognitive alterations (working-memory performance) were compared across groups using linear regression and χ^2^ tests as appropriate. Third, the MLOGIT command was used to fit multinomial logistic regression models with time-lagged variables, while correcting for clustering of data (i.e. two observations for each individual) using the CLUSTER option. In order to test whether the JTC bias contributes to psychosis progression and/or persistence over time, reported symptoms at T1 (categorical variable with five levels), JTC bias (binary variable) and its interaction (psychopathological domains × JTC bias) were added to the model of T2 psychopathological domains as the dependent variable. Relative risk ratios (RRRs) for symptoms progression and persistence were compared using the Wald test. All models were adjusted for various *a priori* defined potential confounders. First, we adjusted for socio-demographic characteristics (i.e. age, sex and level of education) and, subsequently, for cognitive alterations (i.e. working memory performance). Missing values for exposure, outcome or covariates were assumed to be missing at random and, thus, individuals were excluded from statistical modelling, but retained for crude RRR estimates.

## Results

### Basic sample characteristics

In total, the sample consisted of 4618 participants at the third wave. Of these, 4333 completed the beads task, all measures and did not report lifetime prevalence of co-occurring affective dysregulation and frank psychosis at T0, resulting in 8666 observations combining T1 and T2 (93.8%). There were no differences between individuals who completed the beads task and other measures and those who did not with regards to socio-demographic characteristics and cognitive alterations. The sample characteristics are presented in Table 1. As shown, individuals who reported affective dysregulation and/or PEs were slightly younger, female, less educated and had more cognitive alterations. Group differences on variables, including exposure to various socio-environmental risk factors (e.g. childhood trauma, cannabis use, minority status and urbanicity), are reported elsewhere (Radhakrishnan et al., 2019; Reininghaus et al., 2019) and provided in Table 1. Overall, there were considerable differences when individuals with the sole presence of affective dysregulation and co-occurring affective dysregulation and PEs were compared in terms of most sample characteristics, socio-environmental risk factors and cognitive alterations. A frequency table of reported PEs at T1 by specific items is provided in online Supplementary Table S2.

**Table 1.** Basic characteristics of groups derived from 4333 participants over two timepoints

Overall number of observations (*N* = 8666)	Group 1: no symptoms (*N* = 5036)	Group 2: affective dysregulation (*N* = 3138)	Group 3: psychotic experience (*N* = 137)	Group 4: affective dysregulation + aberrant salience^a^ (*N* = 272)	Group 5: affective dysregulation + frank psychosis^a^ (*N* = 83)	Test statistics	*p*
Age (years), mean (s.d.)	50.7 (12.4)	47.9 (12.1)	49.1 (11.7)	47.2 (12.6)	43.6 (11.4)	*F* = 21.70, df = 4	<0.001
Sex, *n* (%)							
Male	2472 (49.1)	1236 (39.4)	56 (40.9)	97 (35.7)	31 (37.3)	χ2 = 86.44, df = 4	<0.001
Female	2564 (50.9)	1902 (60.6)	81 (59.1)	175 (64.3)	52 (62.7)		
Level of education, *n* (%)							
Primary	192 (3.8)	127 (4.1)	7 (5.1)	18 (6.6)	8 (9.6)	χ2 = 56.72, df = 12	<0.001
Lower secondary	1275 (25.3)	780 (24.9)	37 (27.0)	87 (32.0)	27 (32.5)		
Higher secondary	1542 (30.6)	1017 (32.4)	53 (38.7)	104 (38.2)	30 (36.1)		
Higher professional	2027 (40.3)	1214 (38.7)	40 (29.2)	63 (23.2)	18 (21.7)		
Urbanicity, *n* (%)^b^							
Countryside	652 (10.3)	490 (8.5)	23 (10.3)	57 (7.8)	5 (5.7)	χ2 = 40.81, df = 16	0.001
Village (<25k)	2557 (40.3)	2241 (38.7)	92 (41.3)	280 (38.5)	32 (36.4)		
Small city (25k–50k)	820 (12.9)	739 (12.8)	36 (16.1)	91 (12.5)	18 (20.5)		
Medium city (50k–100k)	952 (15.0)	991 (17.1)	30 (13.5)	118 (16.2)	9 (10.2)		
Big city (>100k)	1371 (21.6)	1337 (23.1)	42 (18.8)	181 (24.9)	24 (27.3)		
Minority status, *n* (%)^c^							
Yes	270 (5.4)	207 (6.6)	5 (3.7)	26 (9.6)	8 (9.6)	χ2 = 15.10, df = 4	0.005
No	4766 (94.6)	2931 (93.4)	132 (96.4)	246 (90.4)	75 (90.4)		
Regular cannabis use, *n* (%)^d^							
Yes	8 (0.2)	17 (0.6)	1 (0.7)	2 (0.8)	2 (2.6)	χ2 = 21.86, df = 4	<0.001
No	4940 (99.8)	2992 (99.4)	134 (99.3)	260 (99.2)	74 (97.4)		
Childhood trauma (80th perc.), *n* (%)^e^							
Yes	574 (11.4)	726 (23.1)	28 (20.4)	82 (30.1)	32 (38.6)	χ2 = 261⋅16, df = 4	<0.001
No	4462 (88.6)	2412 (76.9)	109 (79.6)	190 (69.9)	51 (61.5)		
Reasoning bias							
Absent (*n*, %)	2468 (49.0)	1535 (48.9)	63 (46.0)	124 (45.6)	28 (33.7)	χ2 = 9.15, df = 4	0.058
Present (*n*, %)	2568 (51.0)	1603 (51.1)	74 (54.0)	148 (54.4)	55 (66.3)		
Working memory performance^f^							
Mean (s.d.)	20.0 (3.9)	20.2 (3.8)	21.4 (3.6)	21.1 (4.1)	21.7 (3.6)	*F* = 10.27, df = 4	<0.001
Distribution of affective dysregulation across groups (*n*, %)							
Depression	–	2086 (66.5)	–	194 (71.3)	66 (79.5)	–	–
Anxiety	–	2161 (69.0)	–	210 (77.2)	74 (89.2)		
Mania	–	1211 (38.6)	–	126 (46.3)	44 (53.0)		

*Notes*: Data with an overall number of 8666 observations from surveys of 4333 participants who completed all assessments, including the beads task, at two time-points (T1 and T2), excluding those with affective dysregulation + frank psychosis at T0 (*N* = 198).

a Defined as: aberrant salience: low to moderate psychosis levels, one or two PEs; frank psychosis: high psychosis levels, three or more PEs or psychosis-related help-seeking behaviour.

b Defined as exposure to urban environment until the age of 16 years, classified based on Dutch classification data of population density: countryside (large distance to amenities), village (<25 000 inhabitants), small city (25 000–50 000 inhabitants), medium city (50 000–100 000 inhabitants) and larger cities (>100 000 inhabitants).

c Born in any other country than The Netherlands.

d Regular cannabis use was based on the section of Illegal Substance Use from CIDI 3.0. A pattern of use of once per week or more during lifetime (T0) or previous three years (T1, T2) were used as the cut-off.

e Based on sum scores of items asking for five types of childhood trauma before the age of 16: two incidents or more of emotional neglect (i.e. not listened to, ignored or unsupported), physical abuse (i.e. kicked, hit, bitten or hurt with object or hot water), psychological abuse (i.e. yelled at, insulted, unjustly punished, treated, threatened, belittled or blackmailed) or one incidence or more of sexual abuse (i.e. any unwanted sexual experience) and peer victimisation (i.e. bullying). The childhood trauma sum score was dichotomised at the 80th percentile.

f Sum scores of the digit-span task (range 6–30) were recoded that higher numbers indicate lower working memory performance and vice versa.

### JTC bias and psychosis progression and occurrence

As shown in Table 2, compared to individuals with neither affective dysregulation nor PEs at both timepoints, those who reported affective dysregulation and a state of aberrant salience at T1 were more likely, albeit below conventional alpha, to report frank psychosis at T2 if the JTC bias was present [adj. RRR: 3.8, 95% confidence interval (CI) 0.8–18.6, *p* = 0.101]. In contrast, co-occurrence of affective dysregulation and frank psychosis at T2 was not influenced by JTC bias in individuals who reported the sole presence of affective dysregulation at T1 (adj. RRR: 1.3, 95% CI 0.4–5.0, *p* = 0.659).

**Table 2.** Results (RRR and 95% CI) on the association of symptoms at T1 with symptoms at T2 by group and JTC bias

	Model 1^a^		Model 2^b^		Model 3^c^		Model 4^d^	
	RRR (95% CI)	*p*	adj. RRR (95% CI)	*p*	adj. RRR (95% CI)	*p*	adj. RRR (95% CI)	*p*
Reference: no symptoms at T1 and T2								
Outcome: Affective dysregulation + frank psychosis at T2								
Symptoms at T1								
Affective dysregulation	3.3 (1.2–8.7)	0.016	3.3 (1.2–8.7)	0.017	3.1 (1.2–8.2)	0.024	3.1 (1.2–8.1)	0.025
Psychotic experiences	25.7 (7.0–94.8)	<0.001	25.3 (6.8–93.4)	<0.001	23.8 (6.2–91.7)	<0.001	21.7 (5.7–83.2)	<0.001
Affective dysregulation + aberrant salience	9.6 (2.7–34.7)	0.001	9.6 (2.7–34.9)	0.001	8.1 (2.2–29.5)	0.002	7.4 (2.0–27.4)	0.003
Affective dysregulation + frank psychosis	79.2 (6.6–953.8)	0.001	78.0 (6.5–939.8)	0.001	54.1 (5.0–587.5)	0.001	50.5 (5.0–506.4)	0.001
Presence of reasoning bias								
JTC bias	1.1 (0.4–3.3)	0.843	1.1 (0.4–3.3)	0.852	1.2 (0.4–3.7)	0.715	1.2 (0.4–3.5)	0.779
Interaction of symptoms at T1 and reasoning bias								
Affective dysregulation × JTC bias	1.5 (0.4–5.5)	0.535	1.4 (0.4–5.1)	0.632	1.3 (0.4–5.0)	0.658	1.3 (0.4–5.0)	0.659
Psychotic experiences × JTC bias	0.5 (0.1–3.3)	0.509	0.6 (0.1–3.4)	0.525	0.5 (0.1–3.1)	0.453	0.5 (0.1–3.2)	0.474
Affective dysregulation + aberrant salience × JTC bias	4.2 (0.9–20.1)	0.071	3.7 (0.8–17.8)	0.105	3.6 (0.7–17.8)	0.111	3.8 (0.8–18.6)	0.101
Affective dysregulation + frank psychosis × JTC bias	10.7 (0.5–221.3)	0.124	10.8 (0.5–222.6)	0.123	13.0 (0.7–258.9)	0.093	12.7 (0.7–239.6)	0.091

df, degrees of freedom; CI, confidence interval; RRR, relative risk ratio.

a Unadjusted model, unrestricted sample (*N* = 4596 individuals who completed the beads task at the third wave).

b Unadjusted model, restricted sample (*N* = 4333 individuals who completed the beads task as well as other measures).

c Model adjusted for socio-demographics (i.e. age, gender and level of education), restricted sample.

d Model adjusted for socio-demographics and cognitive alterations (i.e. working memory performance), restricted sample.

### JTC bias and psychosis persistence

The presence of the JTC bias was associated, albeit below conventional alpha, with an increased risk to maintain frank psychosis at both timepoints in individuals with lifetime affective dysregulation (Table 2: adj. RRR: 12.7, 95% CI 0.7–239.6, *p* = 0.091). The associations of all other symptoms at T1 with symptoms at T2 by JTC bias are provided in online Supplementary Table S3 and model fit statistics for multinomial logistic regression models in online Supplementary Table S4.

## Discussion

### Main findings

This study investigated whether the JTC bias contributes to psychosis progression and persistence in a community sample. Power was low and although associations were not significant at conventional alpha, we interpret findings at the level of clinical evidence rather than arbitrary statistical cut-off, as recommended recently (Amrhein, Greenland, & McShane, 2019) (i.e. most people would still buy a lottery ticket if the probability of winning was 90% instead of 95%). Thus, there was a suggestion that, compared to those who did not report any symptoms, individuals with lifetime affective dysregulation and a state of aberrant salience at T1 were more likely to report frank psychosis at T2 if the JTC bias was present. Similarly, there was a suggestion that the JTC bias contributed to the persistence of frank psychosis over time in individuals with lifetime affective dysregulation. These associations remained largely unchanged after adjusting for demographics (age, sex and education) as well as for cognitive alterations (working memory performance). However, well-powered prospective cohort studies are needed to replicate these findings.

### Methodological considerations

The unique strength of the current study is that the largest data set on JTC-bias to date was used, for the first time, to prospectively investigate the contribution of the JTC bias on psychosis progression and persistence in a longitudinal representative cohort study. However, the following limitations should be considered before interpreting our findings. First, as presented in Table 3, the number of individuals with lifetime affective dysregulation who progressed from a state of aberrant salience at T1 to frank psychosis at T2 or who reported the persistence of frank psychosis at both timepoints were low (*N* = 20 and *N* = 9, respectively), resulting in imprecise estimates. The null hypothesis significance testing paradigm – and the *p* value threshold intrinsic to it – is currently strongly debated with widely differing views (Hurlbert, Levine, & Utts, 2019; Ioannidis, 2018; McShane, Gal, Gelman, Robert, & Tackett, 2019). Thus, the reported findings should be considered as suggestive but not conclusive evidence. Well-powered longitudinal cohort studies are needed to replicate reported findings. However, this does not preclude, as argued above, inferring valuable insights given marked differences in the presence of JTC bias comparing respective groups (e.g. in 80% of individuals who progressed from aberrant salience at T1 to frank psychosis at T2 the JTC bias was present compared to 54% in those without symptoms at both timepoints). Second, the JTC bias was assessed only once during the study period (T2) and, thus, the presence or the absence of JTC bias was conceptualised as individuals' trait reasoning style. Ideally, the beads task would have been completed at more timepoints for more robust estimates and to take potential fluctuation of reasoning style into account. However, assessment burden was considered to be too high and associated benefits too low, especially considering the findings of low variability of JTC bias over time (So et al., 2012). Importantly, however, an uncontrolled study including 31 help-seeking individuals with psychosis suggests that JTC bias may vary over time and that these changes may be associated with symptom severity (Dudley et al., 2013). Thus, more research is needed that specifically investigates the stability of JTC bias and potential moderators and mediators of change. Third, although we excluded individuals with lifetime affective dysregulation and frank psychosis at T0, we did not exclude all individuals with psychosis (e.g. low/moderate psychosis levels) as resulting stratified groups were considered to be too small to test hypotheses. However, excluding those who have already progressed to high psychosis levels before the study period allowed us to investigate more accurately the role of JTC bias in psychosis progression and persistence as psychosis at T1 and T2 reflect first-time interval occurrence. Fourth, we conceptualised low to moderate levels of psychosis (i.e. individuals who endorsed one or two PEs) to represent a state of aberrant salience and high psychosis levels (i.e. individuals who endorsed three or more PEs or reported psychosis-related behaviour) to reflect frank psychosis. As individuals' level of distress and impairment were not directly considered in constructing scores, it is possible that some individuals with high psychosis levels, especially those who did not seek help from mental health services, did not experience any psychosis-related distress while, conversely, some with low to moderate levels of psychosis may experience distress. This would be at variance with established definitions of anomalous experiences of aberrant salience. Thus, our operationalisation of distinguishing between individuals with aberrant salience and frank psychosis should be interpreted with caution. However, again, the low number of individuals who reported the emergence of help-seeking behaviour between T0 and T1 or T1 and T2 have prevented us from using this more valid indicator for frank psychosis. Fifth, recent evidence suggests that JTC bias may be a manifestation or consequence of general cognitive impairment and may not represent a specific cognitive factor involved in psychosis progression over time (Tripoli et al., 2019). Thus, adjusting for various domains of individuals' cognitive ability would have been preferable. Critically, only working memory performance has been assessed at the second wave (T1) in NEMESIS-2 and used as a proxy for cognitive deficits to minimise assessment burden. In the current study, however, controlling for working memory performance did not attenuate reported associations (see Models 3 and 4 in Table 2). Sixth, PEs investigated in the current study differed in terms of quality and type (e.g. delusional ideations and hallucinations). As there is some evidence that JTC bias may be specific to the development of delusional ideations, investigating this further in sensitivity analyses would have been important. However, after stratification by the types of PEs, group sizes were too small to investigate on specificity of JTC bias in relation to delusional ideations.

**Table 3.** Symptom progression and persistence from T1 to T2 by JTC bias

Symptoms at T1 by JTC bias	Symptoms at T2				
	No symptoms	Affective dysregulation	Psychotic experience	Affective disturbance + aberrant salience^a^	Affective dysregulation + frank psychosis^a^
No symptoms					
JTC bias					
Present (*n*, %)	1476 (51.3)	402 (49.5)	23 (53.5)	19 (57.6)	7 (53.8)
Absent (*n*, %)	1404 (48.7)	410 (50.5)	20 (46.5)	14 (42.4)	6 (46.2)
Affective dysregulation					
JTC bias					
Present (*n*, %)	935 (50.2)	1032 (51.1)	25 (48.1)	67 (53.2)	20 (60.6)
Absent (*n*, %)	927 (49.8)	988 (48.9)	27 (51.9)	59 (46.8)	13 (39.4)
Psychotic experience					
JTC bias					
Present (*n*, %)	60 (61.8)	12 (40.0)	10 (66.7)	3 (30.0)	4 (50.0)
Absent (*n*, %)	37 (38.2)	18 (60.0)	5 (33.3)	7 (70.0)	4 (50.0)
Affective dysregulation + aberrant salience					
JTC bias					
Present (*n*, %)	95 (49.5)	151 (57.2)	15 (62.5)	55 (57.9)	16 (80.0)
Absent (*n*, %)	97 (51.5)	113 (42.8)	9 (37.5)	40 (42.1)	4 (20.0)
Affective dysregulation + frank psychosis					
JTC bias					
Present (*n*, %)	2 (40.0)	6 (50.0)	1 (33.3)	4 (50.0)	8 (88.9)
Absent (*n*, %)	3 (60.0)	6 (50.0)	2 (66.7)	4 (50.0)	1 (11.1)

*Notes*: Data with an overall number of 8666 observations from surveys of 4333 participants who completed all assessments, including the beads task and other measures, excluding those with high psychosis levels at T0 (*N* = 198).

a Defined as: aberrant salience: low to moderate psychosis levels, one to two PEs; frank psychosis: high psychosis levels, three or more PEs or psychosis-related help-seeking behaviour.

### Comparison with previous research

The JTC bias is among the most widely studied cognitive biases in psychosis. However, to our knowledge, no study has prospectively investigated the role of JTC bias in psychosis progression and persistence, testing dominant models of psychosis ontogenesis. The findings of the current study support the suggestion that individuals with JTC bias are more likely to progress from the states of aberrant salience to frank psychosis. Thus, JTC bias may not only cross-sectionally be associated with psychosis liability, as consistently shown (Dudley et al., 2016; Fine et al., 2007; Ross et al., 2015; So et al., 2016), but may also influence the development of more severe psychosis levels or psychosis-related help-seeking behaviour over time. Similarly, the findings support the notion that the JTC bias may contribute to the persistence of frank psychosis. These findings are in accordance with the recently proposed, but rarely tested, models of psychosis which have posited the importance of cognitive factors in the development and maintenance of psychosis (Freeman, 2016; Howes et al., 2017; Howes & Murray, 2014).

Whilst recognising low power and statistically formally inconclusive findings, we hypothesise that if JTC bias indeed contributes to psychosis progression or persistence then, given high rates of JTC bias in individuals without any symptoms, it is likely that JTC bias adds to or combines with other genetic and socio-environmental risk factors. For example, an individual who has been exposed to childhood trauma or developmental hazards early in life may experience otherwise irrelevant stimuli as excessively salient, while, concurrently, risk exposure may have provoked the development of threat beliefs about the world and others (Freeman, 2016). Consequently, in search for an explanation, the initially non-distressing experiences of aberrant salience may be interpreted, as a secondary process, as threatening and externally caused (Freeman, 2016) and, subsequently, lead to more severe psychosis levels and/or the development of help-seeking behaviour. As noted previously (Freeman, 2016), the JTC bias may be particularly important during this stage: individuals' tendency to gather less information to draw conclusions in a standardised cognitive task may translate to real-life situations in the form of hastier decisions about the negative intentions of others and, thus, lowering the probability of processing alternative explanations which may result in stronger delusional convictions. Thus, threat beliefs of salient experiences and associated appraisal processes may be influenced by the presence of JTC bias, especially when combined with low belief flexibility (Ward, Peters, Jackson, Day, & Garety, 2018), another well-established cognitive factor. These processes may give rise to a vicious circle of increasing psychosis severity and distress. However, this has not been directly demonstrated and should be further investigated in future studies. Also, how JTC bias is associated with the behavioural response to psychosis as well as other cognitive factors such as safety-seeking, avoidance, worrying and unhelpful emotional regulation strategies (Freeman, 2016; Garety et al., 2007; Howes & Murray, 2014) should be further investigated.

## Conclusion

There was some evidence that the JTC bias may contribute to psychosis progression and persistence in individuals with lifetime affective dysregulation from the general population. However, large prospective studies are needed to replicate reported findings. An important next step is to further investigate the causal status of JTC bias in the development and maintenance of psychosis in order to inform promising treatment targets (Brown et al., 2019) and develop process-based treatment protocols that aim to directly manipulate reasoning bias and other cognitive factors (Moritz & Woodward, 2007).

## Supplementary material

For supplementary material accompanying this paper visit https://doi.org/10.1017/S0033291720000446. click here to view supplementary material

## References

[ref1] Amrhein, V., Greenland, S., & McShane, B. (2019). Scientists rise up against statistical significance. Nature, 567, 305–307. 30894741 10.1038/d41586-019-00857-9

[ref2] Andreou, C., Treszl, A., Roesch-Ely, D., Kother, U., Veckenstedt, R., & Moritz, S. (2014). Investigation of the role of the jumping-to-conclusions bias for short-term functional outcome in schizophrenia. Psychiatry Research, 218, 341–347. 24836199 10.1016/j.psychres.2014.04.040

[ref3] Brainstorm Consortium, Anttila, V., Bulik-Sullivan, B., Finucane, H. K., Walters, R. K., Bras, J., … Sullivan, P. (2018). Analysis of shared heritability in common disorders of the brain. Science, 360, eaap8757. 10.1126/science.aap8757 PMC6097237 29930110

[ref4] Brown, P., Waite, F., & Freeman, D. (2019). 'Twisting the lion's tail': Manipulationist tests of causation for psychological mechanisms in the occurrence of delusions and hallucinations. Clinical Psychology Review, 68, 25–37. 30617014 10.1016/j.cpr.2018.12.003

[ref5] de Graaf, R., Ten Have, M., & van Dorsselaer, S. (2010). The Netherlands Mental Health Survey and Incidence Study-2 (NEMESIS-2): Design and methods. International Journal of Methods in Psychiatric Research, 19, 125–141. 20641046 10.1002/mpr.317 PMC6878518

[ref6] Dudley, R., Daley, K., Nicholson, M., Shaftoe, D., Spencer, H., Cavanagh, K., & Freeston, M. (2013). 'Jumping to conclusions' in first-episode psychosis: A longitudinal study. The British Journal of Clinical Psychology, 52, 380–393. 24117911 10.1111/bjc.12023

[ref7] Dudley, R., Taylor, P., Wickham, S., & Hutton, P. (2016). Psychosis, delusions and the ‘jumping to conclusions’ reasoning bias: A systematic review and meta-analysis. Schizophrenia Bulletin, 42, 652–665. 26519952 10.1093/schbul/sbv150 PMC4838082

[ref8] Fine, C., Gardner, M., Craigie, J., & Gold, I. (2007). Hopping, skipping or jumping to conclusions? Clarifying the role of the JTC bias in delusions. Cognitive Neuropsychiatry, 12, 46–77. 17162446 10.1080/13546800600750597

[ref9] Freeman, D. (2016). Persecutory delusions: A cognitive perspective on understanding and treatment. The Lancet. Psychiatry, 3, 685–692. 27371990 10.1016/S2215-0366(16)00066-3

[ref10] Freeman, D., & Garety, P. (2014). Advances in understanding and treating persecutory delusions: A review. Social Psychiatry and Psychiatric Epidemiology, 49, 1179–1189. 25005465 10.1007/s00127-014-0928-7 PMC4108844

[ref11] Fusar-Poli, P., Werbeloff, N., Rutigliano, G., Oliver, D., Davies, C., Stahl, D., … Osborn, D. (2019). Transdiagnostic risk calculator for the automatic detection of individuals at risk and the prediction of psychosis: Second replication in an independent National Health Service Trust. Schizophrenia Bulletin, 45, 562–570. 29897527 10.1093/schbul/sby070 PMC6483570

[ref12] Garety, P. A., Bebbington, P., Fowler, D., Freeman, D., & Kuipers, E. (2007). Implications for neurobiological research of cognitive models of psychosis: A theoretical paper. Psychological Medicine, 37, 1377–1391. 17335638 10.1017/S003329170700013X

[ref13] Garety, P. A., & Freeman, D. (1999). Cognitive approaches to delusions: A critical review of theories and evidence. The British Journal of Clinical Psychology, 38(Pt 2), 113–154. 10389596 10.1348/014466599162700

[ref14] Garety, P. A., Kuipers, E., Fowler, D., Freeman, D., & Bebbington, P. E. (2001). A cognitive model of the positive symptoms of psychosis. Psychological Medicine, 31, 189–195. 11232907 10.1017/s0033291701003312

[ref15] Hartmann, J. A., Nelson, B., Ratheesh, A., Treen, D., & McGorry, P. D. (2019). At-risk studies and clinical antecedents of psychosis, bipolar disorder and depression: A scoping review in the context of clinical staging. Psychological Medicine, 49, 177–189. 29860956 10.1017/S0033291718001435

[ref16] Howes, O. D., McCutcheon, R., Owen, M. J., & Murray, R. M. (2017). The role of genes, stress, and dopamine in the development of schizophrenia. Biological Psychiatry, 81, 9–20. 27720198 10.1016/j.biopsych.2016.07.014 PMC5675052

[ref17] Howes, O. D., & Murray, R. M. (2014). Schizophrenia: An integrated sociodevelopmental-cognitive model. Lancet (London, England), 383, 1677–1687. 10.1016/S0140-6736(13)62036-X PMC4127444 24315522

[ref18] Hurlbert, S. H., Levine, R. A., & Utts, J. (2019). Coup de Grâce for a tough old bull: ‘Statistically significant’ expires. The American Statistician, 73, 352–357.

[ref19] Ioannidis, J. P. A. (2018). The proposal to lower *p* value thresholds to 0.005. JAMA, 319, 1429–1430. 29566133 10.1001/jama.2018.1536

[ref20] Kessler, R. C., & Ustun, T. B. (2004). The World Mental Health (WMH) survey initiative version of the World Health Organization (WHO) Composite International Diagnostic Interview (CIDI). International Journal of Methods in Psychiatric Research, 13, 93–121. 15297906 10.1002/mpr.168 PMC6878592

[ref21] Linscott, R. J., & van Os, J. (2013). An updated and conservative systematic review and meta-analysis of epidemiological evidence on psychotic experiences in children and adults: On the pathway from proneness to persistence to dimensional expression across mental disorders. Psychological Medicine, 43, 1133–1149. 22850401 10.1017/S0033291712001626

[ref22] Loberg, E. M., Gjestad, R., Posserud, M. B., Kompus, K., & Lundervold, A. J. (2019). Psychosocial characteristics differentiate non-distressing and distressing voices in 10346 adolescents. European Child & Adolescent Psychiatry, 10, 1353–1363. 10.1007/s00787-019-01292-x PMC6785583 30820670

[ref23] McShane, B. B., Gal, D., Gelman, A., Robert, C., & Tackett, J. L. (2019). Abandon statistical significance. The American Statistician, 73, 235–245.

[ref24] Menon, M., Mizrahi, R., & Kapur, S. (2008). 'Jumping to conclusions' and delusions in psychosis: Relationship and response to treatment. Schizophrenia Research, 98, 225–231. 17897811 10.1016/j.schres.2007.08.021

[ref25] Moritz, S., & Woodward, T. S. (2007). Metacognitive training in schizophrenia: From basic research to knowledge translation and intervention. Current Opinion in Psychiatry, 20, 619–625. 17921766 10.1097/YCO.0b013e3282f0b8ed

[ref26] Nelson, B., McGorry, P. D., Wichers, M., Wigman, J. T. W., & Hartmann, J. A. (2017). Moving From static to dynamic models of the onset of mental disorder: A review. JAMA Psychiatry, 74, 528–534. 28355471 10.1001/jamapsychiatry.2017.0001

[ref27] Peters, E., & Garety, P. (2006). Cognitive functioning in delusions: A longitudinal analysis. Behaviour Research and Therapy, 44, 481–514. 15913544 10.1016/j.brat.2005.03.008

[ref28] Peters, E., Ward, T., Jackson, M., Woodruff, P., Morgan, C., McGuire, P., & Garety, P. A. (2017). Clinical relevance of appraisals of persistent psychotic experiences in people with and without a need for care: An experimental study. The Lancet. Psychiatry, 4, 927–936. 29179936 10.1016/S2215-0366(17)30409-1 PMC5714590

[ref29] Phillips, L. D., & Edwards, W. (1966). Conservatism in a simple probability inference task. Journal of Experimental Psychology, 72, 346–354. 5968681 10.1037/h0023653

[ref30] Radhakrishnan, R., Guloksuz, S., Ten Have, M., de Graaf, R., van Dorsselaer, S., Gunther, N., … van Os, J. (2019). Interaction between environmental and familial affective risk impacts psychosis admixture in states of affective dysregulation. Psychological Medicine, 49, 1879–1889. 30284529 10.1017/S0033291718002635

[ref31] Reininghaus, U., Rauschenberg, C., Ten Have, M., de Graaf, R., van Dorsselaer, S., Simons, C. J. P., … van Os, J. (2019). Reasoning bias, working memory performance and a transdiagnostic phenotype of affective disturbances and psychotic experiences in the general population. Psychological Medicine, 49, 1799–1809. 30160228 10.1017/S0033291718002209

[ref32] Rodriguez, V., Ajnakina, O., Stilo, S. A., Mondelli, V., Marques, T. R., Trotta, A., … Murray, R. M. (2018). Jumping to conclusions at first onset of psychosis predicts longer admissions, more compulsory admissions and police involvement over the next 4 years: The GAP study. Psychological Medicine, 49, 2256–2266. 30392491 10.1017/S0033291718003197

[ref33] Ross, R. M., McKay, R., Coltheart, M., & Langdon, R. (2015). Jumping to conclusions about the beads task? A meta-analysis of delusional ideation and data-gathering. Schizophrenia Bulletin, 41, 1183–1191. 25616503 10.1093/schbul/sbu187 PMC4535629

[ref34] So, S. H., Freeman, D., Dunn, G., Kapur, S., Kuipers, E., Bebbington, P., … Garety, P. A. (2012). Jumping to conclusions, a lack of belief flexibility and delusional conviction in psychosis: A longitudinal investigation of the structure, frequency, and relatedness of reasoning biases. Journal of Abnormal Psychology, 121, 129–139. 21910515 10.1037/a0025297 PMC3283358

[ref35] So, S. H., Siu, N. Y., Wong, H. L., Chan, W., & Garety, P. A. (2016). 'Jumping to conclusions' data-gathering bias in psychosis and other psychiatric disorders – Two meta-analyses of comparisons between patients and healthy individuals. Clinical Psychology Review, 46, 151–167. 27216559 10.1016/j.cpr.2016.05.001

[ref36] StataCorp (2013). StataCorp LP: College Station, TX.

[ref37] Tripoli, G., Quattrone, D., Ferraro, L., Gayer-Anderson, C., Rodriguez, V., Cascia, C. L., … Forti, M. D. (2019). Jumping to conclusions, general intelligence, and psychosis liability: Findings from the Multi-Centre EU-GEI Case-Control Study. bioRxiv 634352. 10.1017/S003329171900357X PMC8020493 32327005

[ref38] van Os, J., & Reininghaus, U. (2016). Psychosis as a transdiagnostic and extended phenotype in the general population. World Psychiatry, 15, 118–124. 27265696 10.1002/wps.20310 PMC4911787

[ref39] Ward, T., Garety, P. A., Jackson, M., & Peters, E. (2019). Clinical and theoretical relevance of responses to analogues of psychotic experiences in people with psychotic experiences with and without a need-for-care: An experimental study. Psychological Medicine, 1–10. 10.1017/S0033291719000576 30944059

[ref40] Ward, T., Peters, E., Jackson, M., Day, F., & Garety, P. A. (2018). Data-gathering, belief flexibility, and reasoning across the psychosis continuum. Schizophrenia Bulletin, 44, 126–136. 28338872 10.1093/schbul/sbx029 PMC5768047

[ref41] Wechsler, D. (1997). WAIS-III: Wechsler adult intelligence scale *(*3rd ed.*)* administration and scoring manual. San Antonio, TX: Psychological Corporation.

[ref42] Wigman, J. T., van Nierop, M., Vollebergh, W. A., Lieb, R., Beesdo-Baum, K., Wittchen, H. U., & van Os, J. (2012). Evidence that psychotic symptoms are prevalent in disorders of anxiety and depression, impacting on illness onset, risk, and severity – implications for diagnosis and ultra-high risk research. Schizophrenia Bulletin, 38, 247–257. 22258882 10.1093/schbul/sbr196 PMC3283146

[ref43] Wusten, C., Schlier, B., Jaya, E. S., Genetic, R., Outcome of Psychosis, I., Fonseca-Pedrero, E., Peters, E., … Lincoln, T. M. (2018). Psychotic experiences and related distress: A cross-national comparison and network analysis based on 7141 participants from 13 countries. Schizophrenia Bulletin, 44, 1185–1194. 29982814 10.1093/schbul/sby087 PMC6192474

