# Supplementary material for: The jumping to conclusions reasoning bias as a cognitive factor contributing to psychosis progression and persistence: findings from NEMESIS-2
Source: Psychol Med. 2020 Mar 16;51(10):1696–703. doi: 10.1017/S0033291720000446 (PMC8327623; doi:10.1017/S0033291720000446)
Supplement: Supplementary file 1 [file S0033291720000446sup.zip › S0033291720000446sup001.docx]

|  | **Table S4.** Model fit statistics for multinomial logistic regression models | | | | | | | | |  |
| --- | --- | --- | --- | --- | --- | --- | --- | --- | --- | --- |
|  | | |  |  |  |  |  |  |  | |
|  | | | **Observations** | **df** | **LL (null)** | **LL (model)** | **AIC** | **BIC** |  | |
|  | | Model 1 | 8666 | 40 | -7816.63 | -7152.92 | 14385.85 | 14668.53 |  | |
|  | | Model 2 | 8666 | 52 | -7816.63 | -7077.14 | 14258.28 | 14625.77 |  | |
|  | | Model 3 | 8666 | 56 | -7816.63 | -7062.12 | 14236.24 | 14632.00 |  | |

*Note:* df, degrees of freedom; LL= Log-likelihood; AIC, Akaike Information Criterion; BIC, Bayesian Information Criterion.
