# Supplementary material for: The jumping to conclusions reasoning bias as a cognitive factor contributing to psychosis progression and persistence: findings from NEMESIS-2
Source: Psychol Med. 2020 Mar 16;51(10):1696–703. doi: 10.1017/S0033291720000446 (PMC8327623; doi:10.1017/S0033291720000446)
Supplement: Supplementary file 1 [file S0033291720000446sup.zip › S0033291720000446sup002.docx]

**Table S1. Items used to measure anxiety, depression, mania, and psychotic experiences**

| **Psychopathological domains** | | |
| --- | --- | --- |
| **Anxiety (CIDI 3**·**0)** | |  |
|  | Item 1 | SC20^a^ “Have you ever in your life had an attack of fear or panic when all of a sudden you felt very frightened, anxious, or uneasy?” |
|  | Item 2 | SC20a ^a^ “Have you ever had an attack when all of a sudden you became very uncomfortable, either became short of breath, dizzy, nauseous, or your heart pounded, or you thought that you might lose control, die, or go crazy?” |
|  | Item 3 | SC26^a^ “Did you ever have a time in your life when you were a “worrier” – that is, when you worried a lot more about things than other people with the same problems as you?” |
|  | Item 4 | SC26a^a^ “Did you ever have a time in your life when you were much more nervous or anxious than most other people with the same problems as you?”  SC26b^a^ “Did you ever have a period lasting one month or longer when you were anxious and worried most days?” |
|  | Item 5 | SC29^a^ “Was there ever a time in your life when you felt very afraid or really, really shy with people, like meeting new people, going to parties, going on a date, or using a public bathroom?” |
|  |  |  |
| **Depression (CIDI 3**·**0)** | |  |
|  | Item 1 | SC21^a^ “Have you ever in your life had a period lasting several days or longer when most of the day you felt sad, empty or depressed?” |
|  | Item 2 | SC22^a^ “Have you ever had a period lasting several days or longer when most of the day you were very discouraged about how things were going in your life?” |
|  | Item 3 | SC23^a^ “Have you ever had a period lasting several days or longer when you lost interest in most things you usually enjoy like work, hobbies, and personal relationships?” |
|  |  |  |
| **Mania (CIDI 3**·**0)** | |  |
|  | Item 1 | SC24^a^ “Have you ever had a period lasting four days or longer when you became so happy or excited that you either got into trouble, people worried about you, or a doctor said you were manic?” |
|  | Item 2 | SC25^a^ “Have you ever had a period lasting four days or longer when most of the time you were very irritable, grumpy, or in a bad mood?” |
|  |  |  |
| **Psychotic experiences**  **(CIDI 1**·**1)** | | |
|  | Item 1 | “people were spying on you” |
|  | Item 2 | „people were following you“ |
|  | Item 3 | “you were secretly being tested on” |
|  | Item 4 | “someone was conspiring against you” |
|  | Item 5 | “a 'double' had taken the place of a loved one” |
|  | Item 6 | “someone was reading your mind” |
|  | Item 7 | “you could hear the thoughts of others” |
|  | Item 8 | “others could hear your thoughts” |
|  | Item 9 | “alien thoughts were placed in your head” |
|  | Item 10 | “someone took thoughts from your head” |
|  | Item 11 | “special messages were sent to you through media” |
|  | Item 12 | “you were influenced by strange energies” |
|  | Item 13 | “you were being controlled by an outer force” |
|  | Item 14 | “your thoughts were being influenced by machines” |
|  | Item 15 | “any other delusion reported by subject” |
|  | Item 16 | “you saw things that no one else could see” |
|  | Item 17 | “you could hear things that no one else could hear” |
|  | Item 18 | “your own thoughts were broadcasted” |
|  | Item 19 | “you smelled strange things, that others could not smell” |
|  | Item 20 | “you had strange sensations, like being touched when no one was around” |

*Note*: Composite International Diagnostic Interview Version, CIDI
^a^ denotations are based on screening items of the CIDI Version 3·0
