# Supplementary material for: The jumping to conclusions reasoning bias as a cognitive factor contributing to psychosis progression and persistence: findings from NEMESIS-2
Source: Psychol Med. 2020 Mar 16;51(10):1696–703. doi: 10.1017/S0033291720000446 (PMC8327623; doi:10.1017/S0033291720000446)
Supplement: Supplementary file 1 [file S0033291720000446sup.zip › S0033291720000446sup003.docx]

**Table S2. Frequencies of reported PEs at T1**

| **Psychotic experiences** | | | **Frequencies  at T1**^a^  **(n, %)** |
| --- | --- | --- | --- |
| **(CIDI 1**·**1)** | | |  |
|  |  | “Since our previous interview,... |  |
|  | Item 1 | ...people were spying on you” | 76 (1.8) |
|  | Item 2 | ...people were following you“ | 21 (0.5) |
|  | Item 3 | ...you were secretly being tested on” | 16 (0.4) |
|  | Item 4 | ...someone was conspiring against you” | 13 (0.3) |
|  | Item 5 | ...a 'double' had taken the place of a loved one” | 1 (0.02) |
|  | Item 6 | ...someone was reading your mind” | 18 (0.4) |
|  | Item 7 | ...you could hear the thoughts of others” | 31 (0.7) |
|  | Item 8 | ...others could hear your thoughts” | 17 (0.4) |
|  | Item 9 | ...alien thoughts were placed in your head” | 10 (0.2) |
|  | Item 10 | ...someone took thoughts from your head” | 4 (0.1) |
|  | Item 11 | ...special messages were sent to you through media” | 5 (0.1) |
|  | Item 12 | ...you were influenced by strange energies” | 1 (0.02) |
|  | Item 13 | ...you were being controlled by an outer force” | 5 (0.1) |
|  | Item 14 | ...your thoughts were being influenced by machines” | 8 (0.2) |
|  | Item 15 | ...any other delusion reported by subject” | 21 (0.5) |
|  | Item 16 | ...you saw things that no one else could see” | 51 (1.2) |
|  | Item 17 | ...you could hear things that no one else could hear” | 27 (0.6) |
|  | Item 18 | ...your own thoughts were broadcasted” | 10 (0.2) |
|  | Item 19 | ...you smelled strange things, that others could not smell” | 35 (0.8) |
|  | Item 20 | ...you had strange sensations, like being touched when no one was around” | 51 (1.2) |

*Note*: Composite International Diagnostic Interview Version, CIDI

^a^ Individuals who reported lifetime prevalence of affective dysregulation and frank psychosis (i.e., more than 3 psychotic experiences or psychosis-related help-seeking behavior) at T0 were excluded from analyses (*N*=198). Thus, reported frequencies represent interval occurrence of psychotic experiences.
