# Supplementary material for: The jumping to conclusions reasoning bias as a cognitive factor contributing to psychosis progression and persistence: findings from NEMESIS-2
Source: Psychol Med. 2020 Mar 16;51(10):1696–703. doi: 10.1017/S0033291720000446 (PMC8327623; doi:10.1017/S0033291720000446)
Supplement: Supplementary file 1 [file S0033291720000446sup.zip › S0033291720000446sup004.docx]

| **Table S3. Results (RRR and 95% CI) on the association of all symptoms at T1 with all symptoms at T2 by group and JTC bias** | | | | | | | | | | | | | | | |  |
| --- | --- | --- | --- | --- | --- | --- | --- | --- | --- | --- | --- | --- | --- | --- | --- | --- |
|  |  | |  | Model 1 ^a^ | |  | Model 2^b^ | | Model 3^c^ | | | | | Model 4^d^ | |  |
|  |  | |  | RRR (95% CI) | p |  | adj. RRR (95% CI) | p |  | adj. RRR (95% CI) | p |  | adj. RRR (95% CI) | | p |  |
|  |  | |  |  |  |  |  |  |  |  |  |  |  | |  |  |
| Reference: no symptoms at T1 andT2 | | | | | | | | | | | | | | | |  |
|  | |  |  |  |  |  |  |  |  |  |  |  |  | |  |  |
| Outcome: Affective dysregulation only at T2 | | | | | | | | | | | | | | | |  |
| Symptoms at T1 | | |  |  |  |  |  |  |  |  |  |  |  | |  |  |
|  | | Affective dysregulation |  | 3·7 (3·2 – 4·3) | <0·001 |  | 3·6 (3·1 – 4·3) | <0·001 |  | 3·5 (3·0 – 4·1) | <0·001 |  | 3·5 (3·0 – 4·1) | | <0·001 |  |
|  | | Psychotic experiences |  | 1·7 (0·9 – 3·0) | <0·001 |  | 1·7 (0·9 – 3·0) | 0·082 |  | 1·6 (0·9 – 2·9) | 0·103 |  | 1·6 (0·9 – 2·8) | | 0·117 |  |
|  | | Affective dysregulation  + aberrant salience |  | 4·0 (3·0 – 5·4) | <0·001 |  | 4·0 (3·0 – 5·4) | <0·001 |  | 3·8 (2·8 – 5·1) | <0·001 |  | 3·7 (2·8 – 5·0) | | <0·001 |  |
|  | | Affective dysregulation + frank psychosis |  | 6·9 (1·7 – 27·6) | 0·007 |  | 6·8 (1·7 – 27·5) | 0·007 |  | 6·0 (1·5 – 24·6) | 0·012 |  | 6·0 (1·5 – 24·4) | | 0·013 |  |
|  | | |  |  |  |  |  |  |  |  |  |  |  | |  |  |
| Presence of reasoning bias | | |  |  |  |  |  |  |  |  |  |  |  | |  |  |
|  | | JTC bias |  | 0·9 (0·8 – 1·1) | 0·430 |  | 0·9 (0·8 – 1·1) | 0·388 |  | 1·0 (0·8 – 1·1) | 0·796 |  | 1·0 (0·8 – 1·1) | | 0·725 |  |
|  | |  |  |  |  |  |  |  |  |  |  |  |  | |  |  |
| Interaction of symptoms at T1 and reasoning bias | | |  |  |  |  |  |  |  |  |  |  |  | |  |  |
|  | | Affective dysregulation x JTC bias |  | 1·1 (0·9 – 1·4) | 0·324 |  | 1·1 (0·9 – 1·4) | 0·341 |  | 1·1 (0·9 – 1·4) | 0·428 |  | 1·1 (0·9 – 1·4) | | 0·427 |  |
|  | | Psychotic experiences x JTC bias |  | 0·47 (0·2 – 1·1) | 0·075 |  | 0·4 (0·2 – 1·0) | 0·060 |  | 0·4 (0·2 – 1·0) | 0·055 |  | 0·4 (0·2 – 1·0) | | 0·057 |  |
|  | | Affective dysregulation + aberrant salience  x JTC bias |  | 1·4 (0·9 – 2·1) | 0·098 |  | 1·5 (1·0 – 2·2) | 0·070 |  | 1·4 (1·0 – 2·2) | 0·082 |  | 1·5 (1·0 – 2·2) | | 0·080 |  |
|  | | Affective dysregulation + frank psychosis  x JTC bias |  | 2·7 (0·3 – 20·9) | 0·352 |  | 1·6 (0·2 – 13·4) | 0·661 |  | 1·7 (0·2 – 14·4) | 0·630 |  | 1·7 (0·2 – 14·5) | | 0·631 |  |
| Outcome: Psychotic experiences only at T2 | | | | | | | | | | | | | | | |  |
|  | | |  |  |  |  |  |  |  |  |  |  |  | |  |  |
|  | | Affective dysregulation |  | 2·1 (1·1 – 3·7) | 0·016 |  | 2·0 (1·1 – 3·7) | 0·016 |  | 2·0 (1·1 – 3·6) | 0·020 |  | 2·0 (1·1 – 3·5) | | 0·022 |  |
|  | | Psychotic experiences |  | 9·6 (3·6 – 25·6) | <0·001 |  | 9·5 (3·6 – 25·2) | <0·001 |  | 9·2 (3·5 – 24·2) | <0·001 |  | 8·4 (3·2 – 22·3) | | <0·001 |  |
|  | | Affective dysregulation + aberrant salience |  | 6·5 (2·9 – 14·7) | <0·001 |  | 6·5 (2·9 – 14·8) | <0·001 |  | 6·1 (2·7 – 13·9) | <0·001 |  | 5·8 (2·5 – 13·3) | | <0·001 |  |
|  | | Affective dysregulation + frank psychosis |  | 47·5 (7·5 – 300·1) | <0·001 |  | 46·8 (7·4 – 295·6) | <0·001 |  | 42·2 (6·6 – 268·2) | <0·001 |  | 39·6 (6·1 – 255·9) | | <0·001 |  |
|  | |  |  |  |  |  |  |  |  |  |  |  |  | |  |  |
| Presence of reasoning bias | | |  |  |  |  |  |  |  |  |  |  |  | |  |  |
|  | | JTC bias |  | 1·1 (0·6 – 2·0) | 0·756 |  | 1·1 (0·6 – 2·0) | 0·771 |  | 1·1 (0·6 – 2·0) | 0·752 |  | 1·1 (0·6 – 1·9) | | 0·856 |  |
|  | |  |  |  |  |  |  |  |  |  |  |  |  | |  |  |
| Interaction of symptoms at T1 and reasoning bias | | |  |  |  |  |  |  |  |  |  |  |  | |  |  |
|  | | Affective dysregulation x JTC bias |  | 0·9 (0·4 – 1·9) | 0·738 |  | 0·8 (0·4 -1·9) | 0·674 |  | 0·8 (0·4 – 1·9) | 0·654 |  | 0·8 (0·4 – 1·9) | | 0·657 |  |
|  | | Psychotic experiences x JTC bias |  | 1·1 (0·3 – 4·0) | 0·883 |  | 1·1 (0·3 – 4·1) | 0·856 |  | 1·1 (0·3 – 4·0) | 0·886 |  | 1·1 (0·3 – 4·1) | | 0·855 |  |
|  | | Affective dysregulation + aberrant salience  x JTC bias |  | 1·5 (0·5 – 4·4) | 0·458 |  | 1·6 (0·5 – 4·5) | 0·419 |  | 1·5 (0·5 – 4·5) | 0·425 |  | 1·6 (0·5 – 4·6) | | 0·412 |  |
|  | | Affective dysregulation + frank psychosis  x JTC bias |  | 0·7 (0·0 – 14·5) | 0·806 |  | 0·7 (0·0 – 14·5) | 0·809 |  | 0·7 (0·0 – 15·2) | 0·828 |  | 0·7 (0·0 – 14·9) | | 0·816 |  |
| Outcome: Affective disturbance + aberrant salience at T2 | | | | | | | | | | | | | | | |  |
| Symptoms at T1 | | |  |  |  |  |  |  |  |  |  |  |  | |  |  |
|  | | Affective dysregulation |  | 6·7 (3·7 – 12·1) | <0·001 |  | 6·4 (3·5 – 11·5) | <0·001 |  | 6·1 (3·4 – 11·0) | <0·001 |  | 6·1 (3·4 – 11·0) | | <0·001 |  |
|  | | Psychotic experiences |  | 19·3 (7·3 – 50·9) | <0·001 |  | 19·0 (7·2 – 50·1) | <0·001 |  | 17·9 (6·6 – 48·5) | <0·001 |  | 16·9 (6·2 – 45·7) | | <0·001 |  |
|  | | Affective dysregulation + aberrant salience |  | 41·1 (21·2 – 79·8) | <0·001 |  | 41·4 (21·3 – 80·3) | <0·001 |  | 36·5 (18·6 – 71·5) | <0·001 |  | 35·2 (17·9 – 69·0) | | <0·001 |  |
|  | | Affective dysregulation + frank psychosis |  | 135·7 (27·8 – 663·7) | <0·001 |  | 133·7 (27·3 – 653·9) | <0·001 |  | 107·2 (23·2 – 495·2) | <0·001 |  | 103·0 (21·5 – 492·9) | | <0·001 |  |
|  | |  |  |  |  |  |  |  |  |  |  |  |  | |  |  |
| Presence of reasoning bias | | |  |  |  |  |  |  |  |  |  |  |  | |  |  |
|  | | JTC bias |  | 1·4 (0·7 – 2·7) | 0·373 |  | 1·3 (0·6 – 2·6) | 0·472 |  | 1·3 (0·7 – 2·6) | 0·443 |  | 1·3 (0·6 – 2·6) | | 0·487 |  |
|  | |  |  |  |  |  |  |  |  |  |  |  |  | |  |  |
| Interaction of symptoms at T1 and reasoning bias | | |  |  |  |  |  |  |  |  |  |  |  | |  |  |
|  | | Affective dysregulation x JTC bias |  | 0·9 (0·4 – 1·9) | 0·720 |  | 0·9 (0·4 – 1·9) | 0·732 |  | 0·9 (0·4 – 1·9) | 0·702 |  | 0·9 (0·4 – 1·9) | | 0·703 |  |
|  | | Psychotic experiences x JTC bias |  | 0·2 (0·0 – 0·9) | 0·039 |  | 0·2 (0·0 – 1·0) | 0·049 |  | 0·2 (0·0 – 1·0) | 0·045 |  | 0·2 (0·0 – 1·0) | | 0·047 |  |
|  | | Affective dysregulation + aberrant salience  x JTC bias |  | 1·0 (0·4 – 2·4) | 0·992 |  | 1·1 (0·4 – 2·6) | 0·853 |  | 1·1 (0·4 – 2·6) | 0·869 |  | 1·1 (0·4 – 2·7) | | 0·847 |  |
|  | | Affective dysregulation + frank psychosis  x JTC bias |  | 1·4 (0·1 – 14·0) | 0·790 |  | 1·2 (0·1 – 12·4) | 0·901 |  | 1·3 (0·1 – 13·6) | 0·835 |  | 1·3 (0·1 – 13·7) | | 0·847 |  |
| Outcome: Affective disturbance + psychosis at T2 | | | | | | | | | | | | | | | |  |
|  | | | | | | | | | | | | | | | |  |
| Symptoms at T1 | | |  |  |  |  |  |  |  |  |  |  |  | |  |  |
|  | | Affective dysregulation |  | 3·3 (1·2 – 8·7) | 0·016 |  | 3·3 (1·2 – 8·7) | 0·017 |  | 3·1 (1·2 – 8·2) | 0·024 |  | 3·1 (1·2 – 8·1) | | 0·025 |  |
|  | | Psychotic experiences |  | 25·7 (7·0 – 94·8) | <0·001 |  | 25·3 (6·8 – 93·4) | <0·001 |  | 23·8 (6·2 – 91·7) | <0·001 |  | 21·7 (5·7 – 83·2) | | <0·001 |  |
|  | | Affective dysregulation + aberrant salience |  | 9·6 (2·7 – 34·7) | 0·001 |  | 9·6 (2·7 – 34·9) | 0·001 |  | 8·1 (2·2 – 29·5) | 0·002 |  | 7·4 (2·0 – 27·4) | | 0·003 |  |
|  | | Affective dysregulation + frank psychosis |  | 79·2 (6·6 – 953·8) | 0·001 |  | 78·0 (6·5 – 939·8) | 0·001 |  | 54·1 (5·0 – 587·5) | 0·001 |  | 50·5 (5·0 – 506·4) | | 0·001 |  |
|  | |  |  |  |  |  |  |  |  |  |  |  |  | |  |  |
| Presence of reasoning bias | | |  |  |  |  |  |  |  |  |  |  |  | |  |  |
|  | | JTC bias |  | 1·1 (0·4 – 3·3) | 0·843 |  | 1·1 (0·4 – 3·3) | 0·852 |  | 1·2 (0·4 – 3·7) | 0715 |  | 1·2 (0·4 – 3·5) | | 0·779 |  |
|  | |  |  |  |  |  |  |  |  |  |  |  |  | |  |  |
| Interaction of symptoms at T1 and reasoning bias | | |  |  |  |  |  |  |  |  |  |  |  | |  |  |
|  | | Affective dysregulation x JTC bias |  | 1·5 (0·4 – 5·5) | 0·535 |  | 1·4 (0·4 – 5·1) | 0·632 |  | 1·3 (0·4 – 5·0) | 0·658 |  | 1·3 (0·4 – 5·0) | | 0·659 |  |
|  | | Psychotic experiences x JTC bias |  | 0·5 (0·1 – 3·3) | 0·509 |  | 0·6 (0·1 – 3·4) | 0·525 |  | 0·5 (0·1 – 3·1) | 0·453 |  | 0·5 (0·1 – 3·2) | | 0·474 |  |
|  | | Affective dysregulation + aberrant salience  x JTC bias |  | 4·2 (0·9 – 20·1) | 0·071 |  | 3·7 (0·8 – 17·8) | 0·105 |  | 3·6 (0·7 – 17·8) | 0·111 |  | 3·8 (0·8 – 18·6) | | 0·101 |  |
|  | | Affective dysregulation + frank psychosis  x JTC bias |  | 10·7 (0·5 – 221·3) | 0·124 |  | 10·8 (0·5 – 222·6) | 0·123 |  | 13·0 (0·7 – 258·9) | 0·093 |  | 12·7 (0·7 – 239·6) | | 0·091 |  |

*Note:* df, degrees of freedom; CI, confidence interval; RRR, relative risk ratio

^a^ Unadjusted model, unrestricted sample (*N*=4596 individuals who completed the beads task at the third wave)

^b^ Unadjusted model, restricted sample (*N*=4333 individuals who completed the beads task as well as other measures)

^c^ Model adjusted for socio-demographics (i.e. age, gender, level of education), restricted sample

^d^ Model adjusted for socio-demographics and cognitive alterations (i.e. working memory performance), restricted sample
